# Prototyping a low-cost open-source autonomous unmanned surface vehicle for real-time water quality monitoring and visualization

**DOI:** 10.1016/j.ohx.2022.e00369

**Published:** 2022-10-13

**Authors:** Jae Hyeon Ryu

**Affiliations:** University of Idaho, United States

**Keywords:** Unmanned surface vehicle (USV), iDroneboat, Water quality, Citizen science, ThingSpeak

## Abstract

A low-cost open-source autonomous unmanned surface vehicle (USV) named “iDroneboat” is developed for real-time monitoring and visualization of water quality. The iDroneboat equipped with Internet of Things (IoT) sensors transmits real-time water quality data, including dissolved oxygen (DO), electronical conductivity (EC), pH, and water temperature (WT) to the cloud for data sharing through Long-term Evolution (LTE) communication protocols. Since material and supplies needed are readily accessible from online marketplaces or local hardware stores, the iDroneboat is easily replicable for local water quality studies and citizen-science activities. The iDroneboat appears to be a promising tool to advance environmental research activities, especially for impaired waterways (*e.g.*, lakes, rivers, and reservoirs). The preliminary result shows that the proposed low-cost platform, iDroneboat, effectively displays water quality components in real-time to the cloud web services (*e.g.*, ThingSpeak), ultimately contributing to citizen science activities and environmental stewardship in water research ecosystems.

## Graphical abstract

**Figure:**
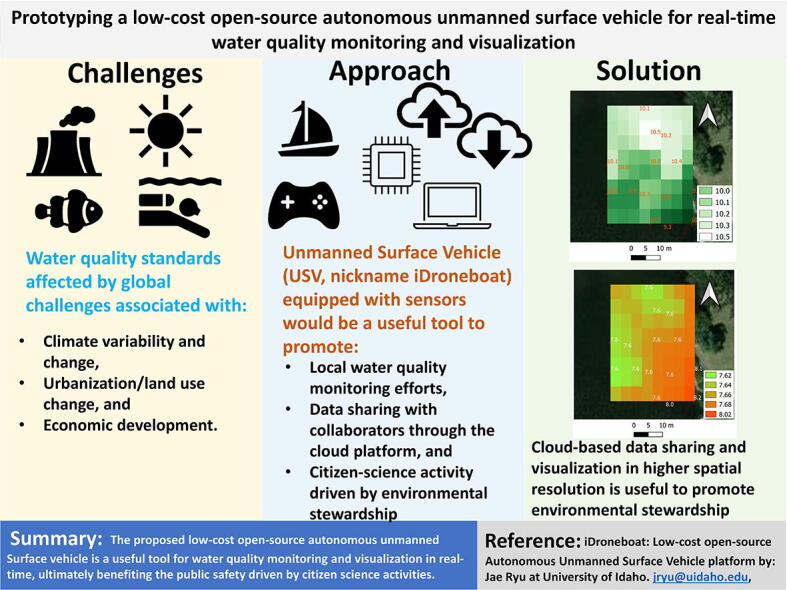

Specifications table

**Table:** 

Hardware name	*iDroneboat*
Subject area	• *Engineering and Material Science* *Educational Tools and Open Source Alternatives to Existing Infrastructure*
Hardware type	• *Field measurements and sensors* *Mechanical engineering and materials science*
Closest commercial analog	*No commercial analog is available*
Open Source License	*Free and open-source software licenses (mainly GPL)*
Cost of Hardware	*$1,557 for iDroneboat*
Source File Repository	https://doi.org/10.17632/fft8c76829.1

## Hardware in context

Environmental issues induced by global challenges, such as climate variability, urbanization, and economic development, continue to renew concerns over the aquatic ecosystem, especially in the complex coupled nature and human interface. A better understanding of environmental consequences is needed to mitigate the associated sectoral ecological impacts. Thus, water quality issues driven by land use change and urbanization continue to highlight water research and sociopolitical agenda at many watershed scales around the world. A better monitoring scheme and water management solution to mitigate such impacts is critical to improving water quality standards at local waterways (e.g., lakes, rivers, reservoirs). A typical practical approach to monitor and tract water condition involves labor-intensive fieldwork, which is very expensive along with safety procedures in open waterbodies. An alternative approach using unmanned surface vehicles (USV) would be important if it provides cost-effective solutions to numerous inland surface waterways and littoral, coastal and offshore problems. The proposed prototype, therefore, could be used widely to contribute to citizen science activities as it improves environmental stewardship in a changing global environment.

Thanks to technological advances, water quality monitoring tools are evolving over time. For example, satellite-based remote sensing technologies, wireless sensor networks (WSN), and automated monitoring stations (AMS) are commonly used as a supplement to the traditional measurement methods of water collection and subsequent laboratory analysis [1]. Additionally, unmanned vehicles, including unmanned aerial vehicles (UAVs) [2], [3], unmanned surface vehicles (USVs) [4], [5], and unmanned underwater vehicles (UUVs) [6], [7] have been also used to monitor water quality components. The recent studies [4], [5] using an USV-based water quality monitoring platform proposed a practical method to measure water quality components, including dissolved oxygen (DO), electronical conductivity (EC), pH, water temperature (WT), and more. For example, one study developed a new, fully open-source, low-cost, and small-sized USV to measure near-surface water quality in real-time in various environments [5], while the other study [4] developed a potential industrial USV to collect garbage on water surface along with water quality monitoring capabilities. Although these two systems are an innovative and new development because it can provide multiple dimensions to facilitate the utility of fast-moving technology on water resources research, those systems are still limited to promote environmental stewardships associated with citizen science activities [8].

Thus, the advantage of the USV platform [5] is a low-cost open-source USV platform for water quality monitoring, but most of moving parts and accessories should be 3-D printed. In contrast, to accomplish its mission associated with water quality monitoring and surface water cleaning jobs, the MF-USV platform [4] has the multi-function capable platform composed of complex system, including a control unit, a locomotion module, a positioning module, an obstacle avoidance module, a water quality monitoring system, a remote human–machine interface, and more. Perhaps, their approach has potential for industrial applications by integrating reliable instruments along with arduous testing and safety protocols. Still, it is not suitable to collect water quality datasets through community-based collaborative citizen science networks. Additionally, both systems might be able to collect water quality data in real-time, but their data sharing protocols are limited to increasing environmental stewardship for broader impacts. This issue has been also highlighted at the professional meetings organized by the American Water Resources Association (AWRA), November 3–6, 2019.

Therefore, the author proposes a prototype of the real-time water quality monitoring and data visualization platform through the cloud-based data sharing protocol, ThingSpeak [9], to fill such a gap, ultimately contributing to community-based citizen science activities for environmental advocacy. Note that the proposed platform is a low-cost open-source USV with real-time data sharing capability and all parts can be obtained from local hardware stores (e.g., homedepot) and/or online marketplaces (e.g., Amazon, eBay) without 3D-printed parts. The preliminary result indicates that the proposed USV-based real-time water quality monitoring and visualization platform could be a promising tool to drive momentum toward enhancing environmental stewardship, ultimately contributing to water research and environmental science for public safety.

## Hardware description

The proposed USV-based water quality monitoring platform (Nickname: iDroneboat) consists of six key components, including 1) control units (Raspberry Pi and Navio2) [10], [11], 2) locomotion module-electrical speed controller (ESC) and propulsion motor, 3) navigation module (global positioning system), 4) water quality sensors [12], 5) communication module (Verizon LTE dongle)[13], and 6) waterproof housing. Raspberry Pi and Arduino are commonly used for many do-it-yourself (DIY) projects because of low-cost, but highly versatile for small projects to larger applications. The Arduino is open-source hardware, and it is initially started at the interaction Design Institute Ivera in Italy around 2003 [14]. The Wiring language can be used to write code for the Atmega-based boards as well as many different Arduino models, including the Arduino Uno which is most popular for DIY projects. Note that the previous studies [4], [5] also use the Arduino board due to its versatility connecting nearly unlimited sensor nodes through the extended general-purpose input/output (GPIO) modules. Although there are many Arduino-based applications in real-world scales, such as a remote-controlled (RC) car, boat, and airplane, Arduino itself is not considered as a computer, but a general-purpose microcontroller. By contrast, Raspberry Pi is a low-cost, credit-card sized tiny computer that plugs into a computer monitor or television and uses a standard keyboard and mouse. It can interact with the outside world and has been used in a wide array of digital maker project from small to larger tasks by integrating multiple sensors (e.g., infra-red camera) and accessories [11]. Since the most recent version of the Raspberry Pi with built-in Wi-Fi module (Raspberry Pi Version 4) can transmit the collected data from the field to the cloud via Long-Term Evolution (LTE) communication module, so many IT industries, such as Sixfab [15] have devoted to developing an add-on module for Raspberry pi. Based on the previous study [3], the proposed iDroneboat also uses a similar setup for convenience.

In addition to Raspberry Pi, Navio2 [10], an autopilot HAT for Raspberry Pi is also used to accomplish autonomous missions powered by Ardupilot and Robot Operating System (ROS). Navio2 is a flight controller that offers all I/O needed for a flight controller being used for the Raspberry Pi board’s HAT module [16]. Expanded processing power and data acquisition with a combination of Raspberry pi and of Navio2 together enable higher capacity to build a powerful low-cost iDroneboat.

The proposed iDroneboat could contribute to USV-based water quality studies in the sense that it can monitor water components at multiple points by navigating open waterbodies, ultimately benefiting the water research community in the following areas.

• water quality monitoring and visualization through the cloud-based data-sharing platform to increase environmental stewardship at watershed scales
• Water quality studies at inland waterbodies (*e.g.*, lakes, rivers, reservoirs)
• Citizen science activities for the public safety at local watersheds.

## Design files

As briefly mentioned above, the proposed iDroneboat doesn’t require 3D printed files to build. All parts can be purchased from local hardware stores or online marketplaces, so no design files are needed to replicate another iDroneboat. A set of parameters for iDroneboat and R codes for geotagging and visualization process are available on the online depository at Mendeley Data (https://doi.org/10.17632/fft8c76829.1).

## Bill of materials summary

The complete bill of materials (BOM) to replicate the iDroneboat alongside the selection of sensors implemented is listed in Table 1. The listed components are not highly specialized and can be purchased from various local and online marketplaces, such as Amazon.

**Table 1:** iDroneboat Bill of Materials*.*

Designator	Component	Number	Cost per unit-currency	Total cost-currency	Source of materials	Material link
Main Controller	Raspberry Pi 4 Model B (RP4)	1	$144.99	$144.99	Online Store	https://amz.run/5H8t
	NAVIO2	1	$144.99	$144.99	Online Store	https://bityl.co/AkWk
	Radio Telemetry Kit	1	$28.99	$28.99	Online Store	https://amz.run/5HpD
	6 s 22.2 V Lithium Polymer (LiPo) battery	1	$32.49	$32.49	Online Store	https://amz.run/5Hpj
	Taranis Remote Controller (RC)	1	$229.00	$229.00	Online Store	https://amz.run/5HqT
	Taranis RC Receiver	1	$39.95	$39.95	Online Store	https://amz.run/5HqW
Locomotion Module	Propulsion: 12 V underwater thruster brushless motors (CW&CCW)	1	$63.99	$63.99	Online Store	https://amz.run/5vKm
	REC 30A ESC UBEC	1	$8.49	$8.49	Online Store	https://amz.run/5H8x
	6 s 22.2 V Lithium Polymer (LiPo) battery	2	$32.49	$64.99	Online Store	https://amz.run/5Hpj
Positioning Module	Waterproof GPS Navigation with MCS connector (∼3 m) 3–5 V DC	1	$9.99	$9.99	Online Store	https://amz.run/5H8z ??
Water Quality Sensing Module	Sensor kit	1	$499.99	$499.99	Online Store	https://bityl.co/AkXJ
	1S 3.7 V Lithium Polymer (LiPo) battery	1	$33.82	$33.82	Online Store	https://amz.run/5Hpi
LTE communication module	Verizon LTE Global USB Modem*	1	$151.99	$151.99	Online Store	https://amz.run/5H94
Cabling and Housing Boxes	Plastic box (17 Liter)	1	$16.99	$16.99	Online or local Staples store	https://bityl.co/AkXW
	Plastic box (3 Liter)	1	$6.99	$6.99	Online or local Staples store	https://bityl.co/AmbD
	Plastic box (1.5 Liter)	1	$10.74	$10.74	Online or local Staples store	https://amz.run/5Hes
	Waterproof Switch 12 V 20A	2	$1.90	$3.8	Online Store	https://amz.run/5H9O
	Water resistant cable gland	1	$15.98	$15.98	Online Store	https://amz.run/5Hp6
	Rubber washer	1	$11.99	$11.99	Online Store	https://amz.run/5HqK
	Sealing strip	1	$17.88	$17.88	Online Store	https://amz.run/5HqM
Miscellaneous parts	M2.5 screws set	1	$14.99	$14.99	Online Store	https://amz.run/5Hoh
	Wi-Fi dongle	1	$14.99	$14.99	Online Store	https://amz.run/5Hom
	Clear PVC tubing hose	1	$10.89	$10.89	Online Store or local hardware store (e.g., homedepot)	https://amz.run/5Hp5
	Power module	1	$12.50	$12.50	Online Store	https://amz.run/5VRE

*Subscription is required to activate this model through wireless service provider (*e.g.*, Verizon Wireless).

## Build instructions

### Hardware setup

**Fig. 1:**
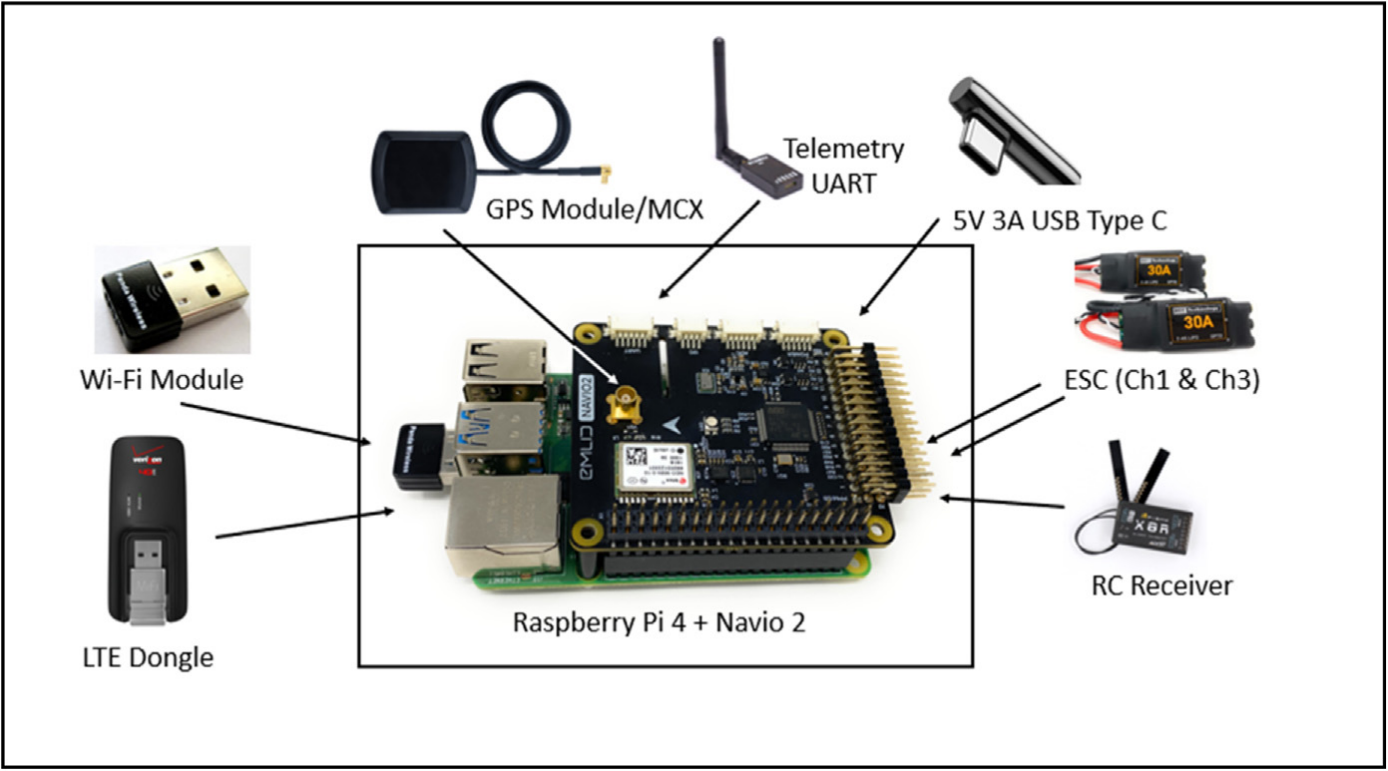
Hardware architecture of the main controller (Raspberry Pi 4 + Navio 2).

To accomplish autonomous navigation missions, Navio 2 was used as the main controller. Navio 2 is a flight controller used for drones by integrating various sensors onboard [10]. As an autopilot shield (or hat) for Raspberry Pi (RP), it is easy to install on the top of the RP board (See Fig. 1.). To tight two devices (RP and Navio 2) securely, M2.5 screw and 10 mm spacer (or standoff) are needed to ensure reliable internal communications within I/O channels. Fig. 1. Shows a basic hardware architecture of the central controller composed of RP and Navio 2.

A pair of Electronic Speed Controller (ESC) is also needed to operate two propulsion motors −1 clockwise (CW) and 1 counterclockwise (CCW) by converting a single signal from Navio 2 to a high power pulse [17]. Although Raspberry Pi Version 4 (RP4) has a built-in Wi-Fi module as a primary network device (wlan0), another Wi-Fi device (e.g., USB dongle) was used as the secondary Wi-Fi interface (wlan1) to transmit data from Internet Of Thing (IoT) sensors to RP4. Thus, wlan0 acts as a Wi-Fi access point (or hotspot) to communicate over the internet, while wlan1 is a client with a dynamically assigned internet protocol address (dynamic IP address) to transmit IoT data via the internal Wi-Fi networks. The advantage of this setup is easily scalable to collect numerous datasets from multiple IoT sensors (e.g., color sensor, ultrasonic sensor, an optical sensor) by establishing a wireless communication link with the attached wlan1 to RP4 when needed. To collect real-time water quality data, including DO, EC, pH, WT, the Wi-Fi sensing board (SB) built-in Adafruit Huzzah32 (CPU), ESP32 communication module, and probes were used [12].

**Fig. 2:**
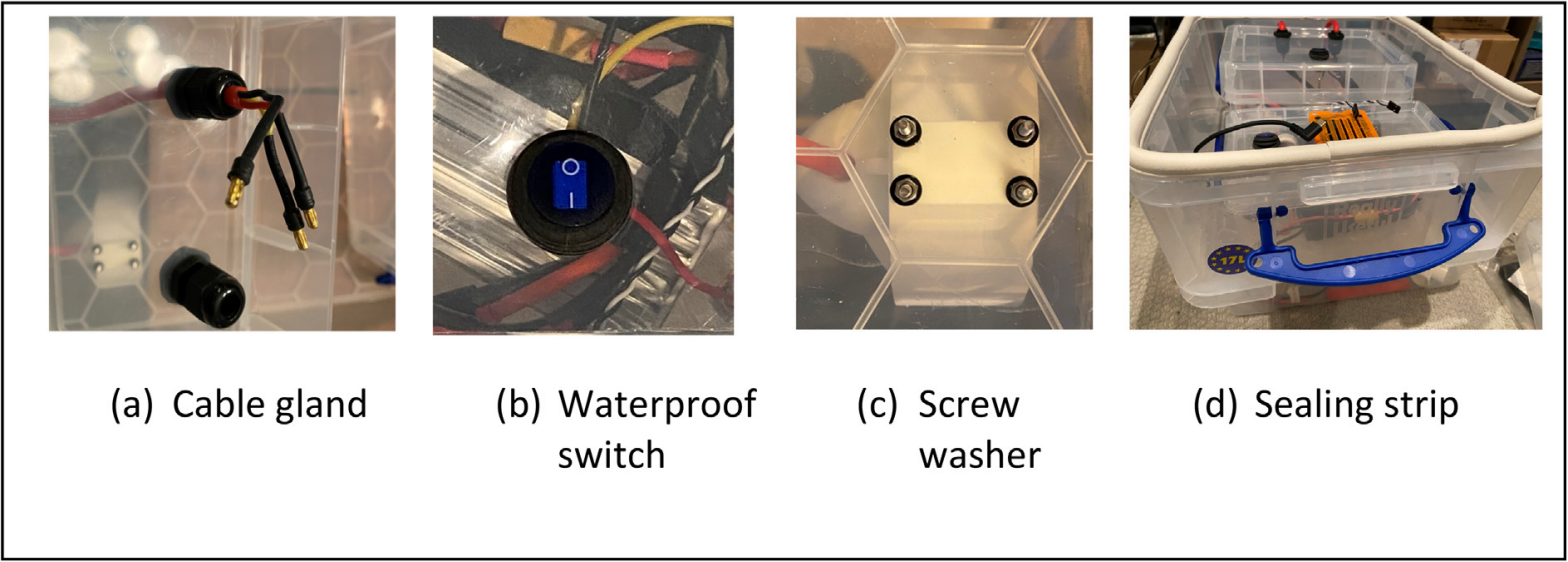
Waterproof housing and cabling to avoid water intrusion; (a) cable gland, (b) waterproof switch, (c) screw washer, and (d) sealing strip.

**Fig. 3:**
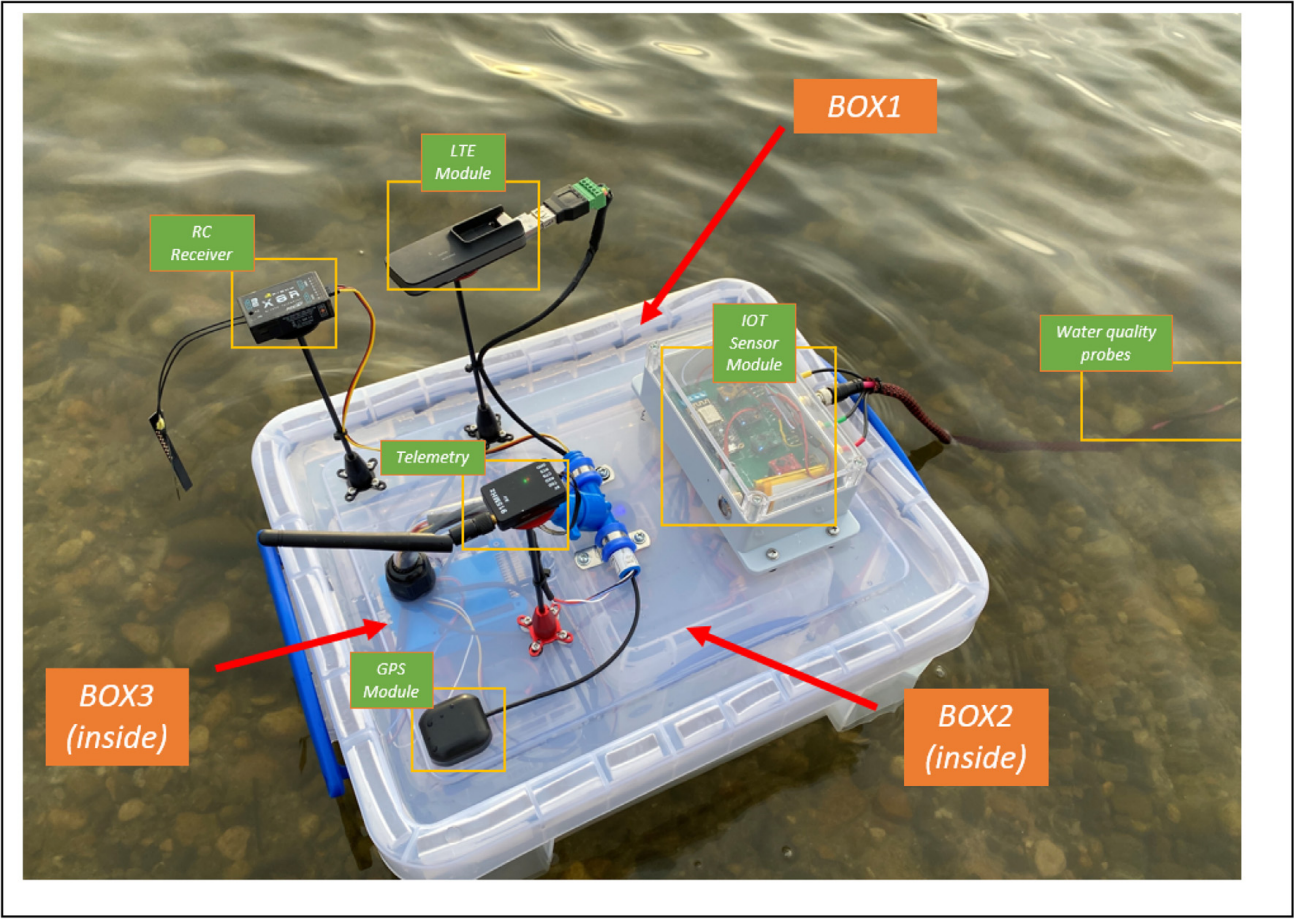
Overview of the iDroneboat.

Additionally, a cellular IoT USB dongle was plugged in RP4 to transmit in-situ water quality data to the cloud data sharing portal [9] via LTE communications. Unlike the hardware setup used in the previous study for airborne UASWQP [3], a USB dongle was used instead for this waterborne experiment because the cellular device attached to RP4 should be deployed outside the waterproof housing control box to strengthen communication signals, while RP4 itself should be enclosed inside the box to prevent water damages. Although Navio 2 can be purchased with a Global Positioning System (GPS) antenna as optional, about 2-meter length cables are recommended to secure slack room while wiring tasks easily through a clear 12-mm outer diameter PVC tubing hose along with a water-resistant cable gland (See Fig. 2, Fig. 3). A set of 3DR Radio telemetry kit with 915 Mhz 500 MW listed in Table 1 was also used to establish communication between Navio 2 and the open-source ground control software (GCS), Mission Planner [18] by transmitting Proportional-Integral-Derivative (PID) signals through the Universal asynchronous receiver-transmitter (UART) interface [19] (see Fig. 1).

### Battery configuration

**Fig. 4:**
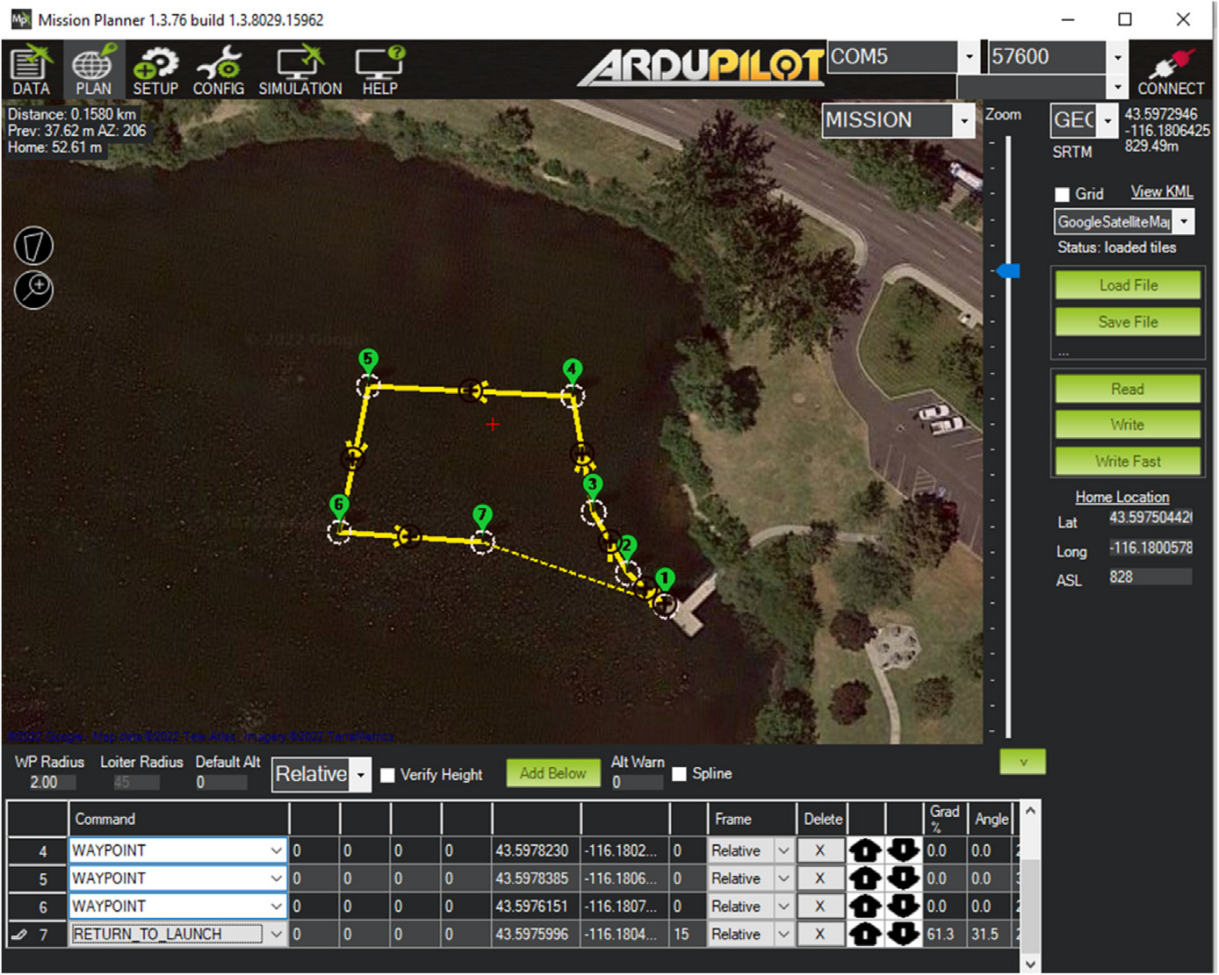
An example of the navigation mission using Mission Planner [18].

The iDroneboat is powered by multiple batteries to supply sufficient power for battery-powered devices, including RP4, ESC/motors, and IoT sensors. The most significant power source needed for iDroneboat is for the locomotion module equipped with ECS and propulsion motors, so a jumper cable was used wiring two batteries in parallel (BAT1) by connecting both the positive terminals. Note that additional batteries can be added to the parallel battery configuration to maintain the same capacity rating for longer operational projects when needed. Another set of batteries is recommended to supply power for the central controller composed of RP 4 and Navio 2. Although power for Navio 2 can be withdrawn by a typical power module (see Table 1) from BAT1, a separate set of batteries is recommended to supply the power source to Navio 2 through RP4. Thus, another 6 s Lithium Polymer (LiPo) battery (BAT2) was used to supply constant power for RP4 separately from BAT1 by minimizing power interrupt induced by rapid thrust actions while navigating. Since the 6S LiPo battery provides a nominal output voltage of 22.2 V which varies from 25.2 V when fully charged, down to 19.2 V, where upon the internal battery regulator disables the battery from over discharge, a voltage regulator was used to provide 3A with 5.1 V DC power for the target value. The power requirement for the IoT sensors is relatively minimal so a 3.7 V 2000mAh LiPo battery (BAT3) was used. Unlike BAT1 and BAT2, BAT3 is very light and small size (80 mm length × 38 mm width × 7.87 mm height) so that it can be inserted into the IoT sensor box housing (19 cm length × 9 cm width × 6 cm height) (see Fig. 4).

### Waterproof housing

Basically, a 17-liter plastic box (BOX1) can be a floating object due to an upward buoyant force computed by multiplication of the density of the displaced fluid (e.g., water), the submerged volume of the object, and gravity. That being said, BOX1 can float in water by accommodating roughly 1000 cc volume (equivalently 10 kg), including all materials described above. The total weight of iDroneboat is measured 5.9 kg so its floating action while navigating was easily justified with 5 km/hr cruise speeds. However, proper waterproofing with cable glands listed in Table 1 is required to avoid water intrusion into BOX1. Additionally, necessary efforts are needed to install waterproof switches for power controls on a 3-liter plastic box (BOX2) and a 1.5-liter plastic box (BOX3) that both are situated inside BOX1. These switches are very convenient to shutdown RP4 and shut off battery connections in an emergency. Although propulsion motors come with 3-mm mounting screws, proper washers should be used for sealing water (See Fig. 2). Lastly, a sealing strip can be also used for extra protection, although all electronic parts are enclosed in the boxes (BOX1, BOX2, and Box3) with lids and a water-avoidance setup.

A complete set up is shown in Fig. 3.

## Operation instructions

### Calibration

There are three different calibration tasks required before launching the iDroneboat. First, the operator should calibrate ESC to ensure that the remote controller sends a feasible range (e.g., minimum and maximum) of Pulse Width Modulation (PWM) values to activate ESC-driven motors. A typical procedure begins with binding tasks to establish communications between the remote controller (RC) and its receiver listed in Table 1. Once the binding is complete, plug ESC three-wire (e.g., positive, negative, and signal) or two-wire (e.g., negative and signal) cables into the throttle channel, which is channel 3 on the RC receiver. An orientation of ESC three-wire/two-wire cables depends on ESC manufacturers but be sure the signal cable should be properly positioned. Next, basic accelerometer calibration and compass calibration using Mission Planner [18] are required to interpret physical orientational information to electrical characteristics, such as magnetic sensitivity, transverse sensitivity, frequency responses, and accurate geographic positions. Lastly, calibration efforts should be made to calibrate IoT water quality sensors based on the manual before the probes contact water [12].

### Mission plan

There are many ground control software (GCS) available at the market to create an autonomous mission for the iDroneboat. A brief description of commonly used GCS is listed in Table 2.

**Table 2:** List of commonly used ground control software (GCS) for autonomous vehicles.

Name	Skill levels	Software Platform	Price (USD)	Links
Autopilot	Medium	Mac OS (iPhone/iPad)	$29.99	https://bityl.co/AnUc
DJI Go	Easy	Mac OS and Android	Free	https://bityl.co/AnUh
Litchi	Medium	Mac OS/Android/Windows	$22.99	https://bityl.co/AnUm
Mission Planner	High	Windows/Linux	Free, open-source	https://bityl.co/AnUv
Pix4DCaputure	Easy	Mac OS and Android	Free	https://bityl.co/AnV8
Qgroundcontrol	Medium	Windows/Linux/Android	Free, open-source	https://bityl.co/AnUy
UGCS	High	Windows/Linux/Mac OS/Android	Subscription basis ($39– $2000)	https://bityl.co/AnVA

The Mission Planner [18] is used for this study not only because it is free and open-source software but also it was used for many other applications due to its versatility and utility. Two key parameters should be set properly in order to control the iDroneboat’s directional motions (e.g., forward and steering movements) on the water surface. Thus, SERVO1_FUNCTION and SERVO3_FUNCTION in MP should be set to 73 (Throttle Left) and 74 (Throttle Right), respectively to change moving directions by varying the speed of two independent propulsion motors (CW&CCW). A navigation mission can be easily created using tools available in MP and a full set of parameters is available at the depository described in the section, Design Files.

### Operation mode

Three operation modes, including AUTO, MANUAL, and RETRUN TO LAUNCH (RTL) are employed to operate the iDroneboat safely. Basically, the iDroneboat navigates autonomously to complete its mission, but it would be useful to change the course of the mission in case of an emergency. For example, when the iDroneboat is interrupted by wildlife (e.g., duck, bird, beaver) nearby, the best operational strategy should be in place to avoid any complications of such consequences. The operator should take a control over the iDroneboat with the RC in MANUL model. It is not uncommon that other autonomous vehicles or large vessels nearby could cause other damage. RTL mode can initiate the autonomous vehicle heading back to the home where it launched to prevent further incidents. Using a 3-position switch available on the RC, three operation modes can be assigned by enabling each switch mix to be source of the PWM range with an available channel (e.g., Channel 5).

**Fig. 5:**
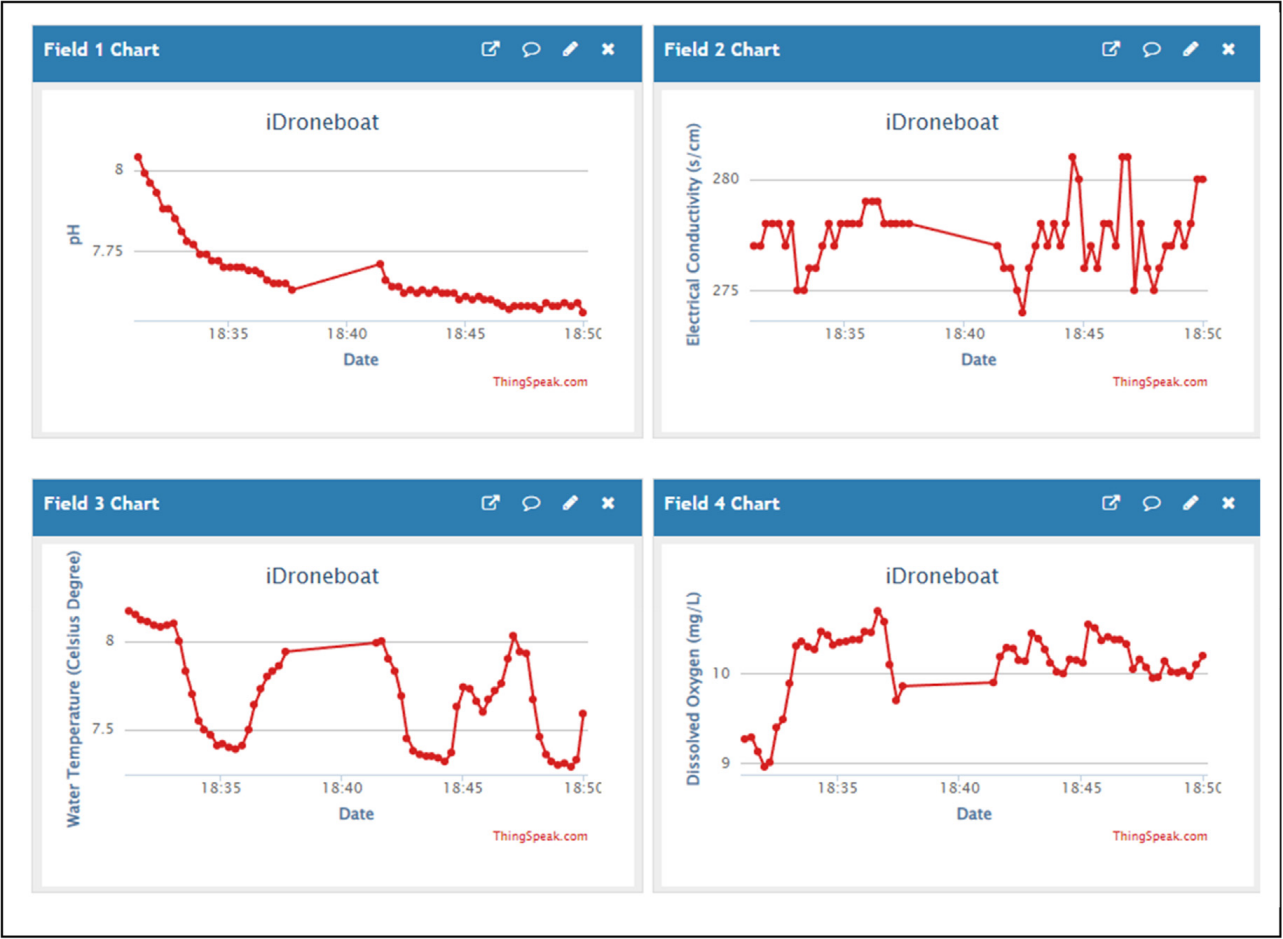
An example of ThingSpeak’s Graphical User Interface shows water quality data (pH, water temperature, EC, and DO).

The iDroneboat was launched from the shore by hand and started navigation from the set home point (e.g., Waypoint 1) manually (MANUAL mode). Once the iDroneboat takes off and waited for the next command in idle status, the operator can verify the overall surrounding status, including potential obstacles and/or hazards nearby for the safe navigation. After a visual check is complete, the AUTO mode can be activated by toggling from the MANUAL mode to enable the iDroneboat to navigate to the next waypoint in the mission. While autonomous navigation is in progress, IoT sensors collect in-situ water quality data (DO, EC, pH, and WT) from the water surface and send it to ThingSpeak, a cloud-based data-sharing platform. ThingSpeak is an open-source IoT analytics platform service that allows aggregating, visualizing, and analyzing real-time data streams in the cloud [9]. Thus, water quality data measured by IoT probes [12] are transferred to the flexible ThingSpeak cloud service via the Verizon LTE module [13] for data visualization. Fig. 5 shows an example of ThingSpeak’s graphical user interface (GUI) from a web browser’s dashboard (Web). The author has assigned four water quality components (DO, EC, pH, and WT) for the first, second, third, and forth field, respectively. Fig. 5 shows the relationship between each water quality component and time, where changes are updated in 15-second intervals. Here, the average pH value is recorded at about 7.5 after the first few minutes for idle conditions and sensor stabilization. Likewise, through the web browser’s interface, additional visualization can be achieved using mobile apps (e.g., ThingView in Apple iPhone)(Not shown in this paper). A data collection in action is available at Youtube [20].

## Validation and characterization

**Fig. 6:**
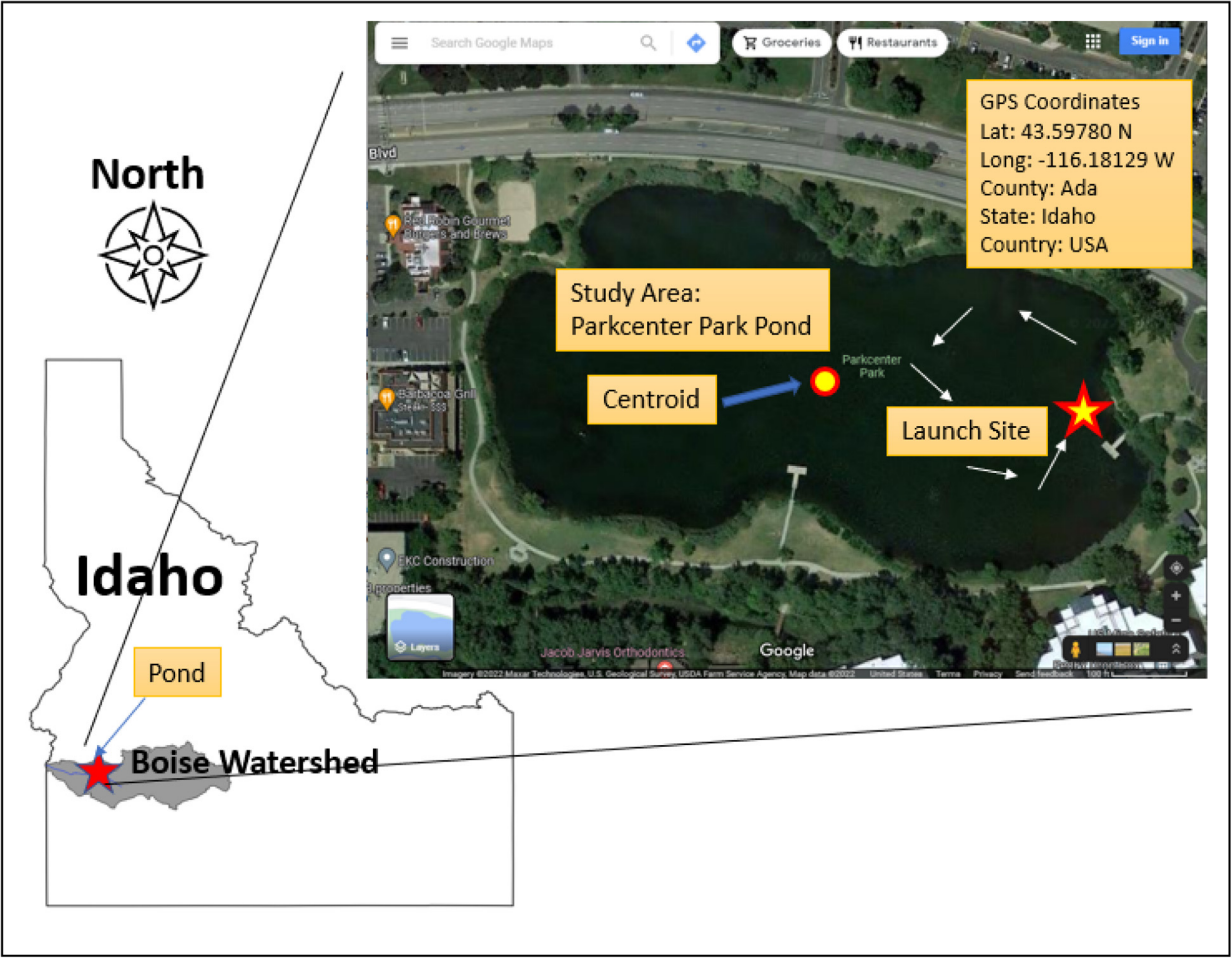
A study area in Parkcenter Park located in Boise, Idaho.

To demonstrate water quality mapping using the collected data (DO, EC, pH, and WT) from in-situ real-time IoT sensors, an autonomous navigation mission took place at a small pond belonging to Parkcenter Park (Coordinates; Latitude:43.5978, Longitude: −116.1810) located in Boise, Idaho USA. Parkcenter Park itself [21]. Although data collected from the IoT sensors are available online via ThingSpeak and stored in a micro-SD card, no location information is recorded in meta format. Thus, an additional step is needed to geotag those data by extracting coordinates and timestamps from the GPS module. To do a pre-processing task for data visualization, a quick and dirty code using R software is available in the data repository described in the section, Design Files (see Fig. 6).

**Fig. 7:**
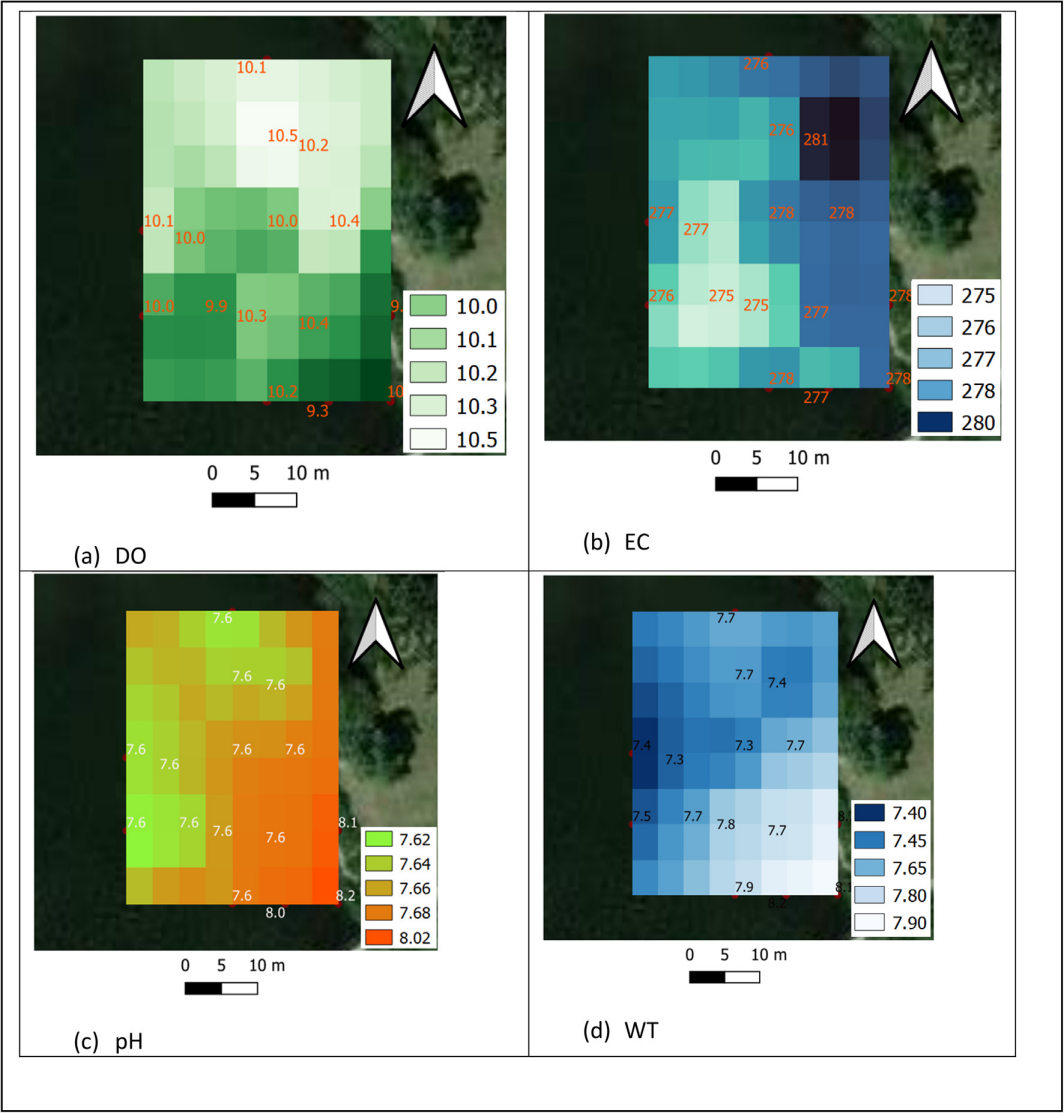
Visualization map of water quality data, including (a) DO, (b) EC, (c) pH, and (d) WT.

To visualize water quality data briefly for the demonstration purpose, Qgis, a free and open-source geographic information system (GIS) software was also used. A built-in inverse distance weighted (IDW) tool was utilized to illustrate spatial representation of individual water component as shown in Fig. 7.

## Summary and future work

A low-cost open-source unmanned surface vehicle (USV; nickname iDroneboat) was developed to monitor and visualize in-situ water quality data, including DO, EC, pH, and WT in real-time. Using low-cost materials available in the marketplace (e.g., Amazon online store or local hardware stores), a prototype of the iDroneboat can be easily built to collect water quality (WQ) data at open waterbodies (e.g., lakes, reservoirs). Thanks to technological advances, the iDroneboat equipped with GPS, sensors, and autonomous capabilities, performed very well in collecting and visualizing data through the cloud-based data sharing platform, ThingSpeak [9].

Although the proposed prototype performed well in conducting data monitoring activities on small pond scales, the iDronebot isn’t necessarily a solid platform to be deployed in larger open waterbodies, such as Lake Michigan or Ocean due to the communication limitation. As briefly stated above, the operator can retrieve the iDroneboat in case of an emergency caused by wildlife nearby or potential system fault. Under the circumstance, a retrieval action plan should be in place. For example, another set of propulsion system should be added to retrieve the vehicle in the case that one of the propulsion motors has a failure possibly driven by a mechanical fault or water intrusion associated with water activities (e.g., swimming, boating) nearby. To minimize such a mishap, additional efforts should be made to attach necessary parts to be used to execute rescue missions easily by a kayaker, other boats, or through the unmanned aerial vehicle (UAV)’s uplift task. Improving potential failsafe capabilities may also minimize unprecedent risks associated with navigation hazards. Additionally, the fifth-generation cellular networks (5G) beyond LTE communications would be a promising solution to establish stable data streaming as well as real-time control in case of an emergency. Developing a USV swarm algorithm (group missions with multiple vehicles) equipped with various sensors (e.g., collision avoidance sensor) and 5G capabilities would also be useful to generate WQ maps over large waterbodies. To enhance endurance navigation, renewable energy, such as solar and/or wind power could be used as a supplementary energy source along with hydrogen fuel cell or hybrid.

Lastly, to improve visualization for larger projects, enterprise- scale web services, such as Amazon Web Services could be considered to ensure deliverables online seamlessly, ultimately promoting environmental stewardships for the public safety.

## Financial support

The author would like to acknowledge Idaho Water Resources Research Institute (IWRRI) for providing USGS 104B grant support for this study. J.H.R is supported partially by the National Institute of Food and Agriculture, U.S. Department of Agriculture (USDA), under ID01507. Any opinions, findings, conclusions, or recommendations expressed in this publication are those of the authors and do not necessarily reflect the view of USGS and USDA.

## Declaration of Competing Interest

The authors declare that they have no known competing financial interests or personal relationships that could have appeared to influence the work reported in this paper.
